# Remembering More Jewish Physicians

**DOI:** 10.5041/RMMJ.10253

**Published:** 2016-07-28

**Authors:** George M. Weisz, Andrzej Grzybowski

**Affiliations:** 1 School of Humanities (Program in History of Medicine), University of New South Wales, Sydney, Australia; 2 School of Humanities, University of New England, Armidale, New South Wales, Australia; 3 Department of Ophthalmology, Poznan City Hospital, Poznań, Poland; 4 Department of Ophthalmology, University of Warmia and Mazury, Olsztyn, Poland; 5 Editor-in-chief, Archives of the History and Philosophy of Medicine, Poznań, Poland

**Keywords:** Bacteriophage, Holocaust, lathyrism, medical heroism

## Abstract

The history of medicine has been an intriguing topic for both authors. The modern relevance of past discoveries led both authors to take a closer look at the lives and contributions of persecuted physicians. The Jewish physicians who died in the Holocaust stand out as a stark example of those who merit being remembered. Many made important contributions to medicine which remain relevant to this day. Hence, this paper reviews the lives and important contributions of two persecuted Jewish physicians: Arthur Kessler (1903–2000) and Bronislawa Fejgin (1883–1943).

## INTRODUCTION

While in theory we understand the importance of history, in practicality there is a tendency to ignore it. This can lead to important discoveries being overlooked, minimized, or wrongly credited. This is particularly likely to happen when little is known about the person who did the original research. However, the rich tradition of Western medicine, which so proudly dates back to the Hippocratic oath, does itself and the future a great disservice if the past is not remembered.

The inspiration for this paper was Dr Israel Milejkowski, Chief Medical Officer of the Warsaw Ghetto. Before his untimely demise on the way to Treblinka in 1943, he said:

And you, Jewish physicians, you deserve some words of recognition. What can I say to you, my companions in misfortune?^1^

Milejkowski’s “companions in misfortune” surely merit being remembered. Indeed, a number of reviews of persecuted physicians have been published, mostly in Israeli journals.^1^–^8^ In light of modern advances related to lathyrism in starving populations, and the potential use of biophages for antibiotic-resistant bacteria, two such physician-researchers and their work merit our attention.

This paper reviews the lives and work of two physician-researchers whose contributions to the above-mentioned fields have either been underestimated or ignored—Dr Arthur Kessler and Dr Bronislawa Fejgin. Dr Kessler’s impact on medicine came about because of the horrors of the Holocaust, whereas Dr Fejgin’s impact has been nigh forgotten for the same reasons. Nevertheless, their contributions to medical research are of great relevance today and will remain as their personal legacy to modern medicine.

## ARTHUR KESSLER (1903–2000)

**Figure 1:**
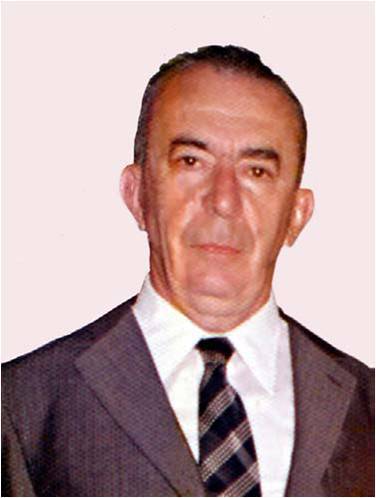
Portrait of Dr Arthur Kessler. Source: From the private collection of D. Kessler, with permission.

**Figure 2:**
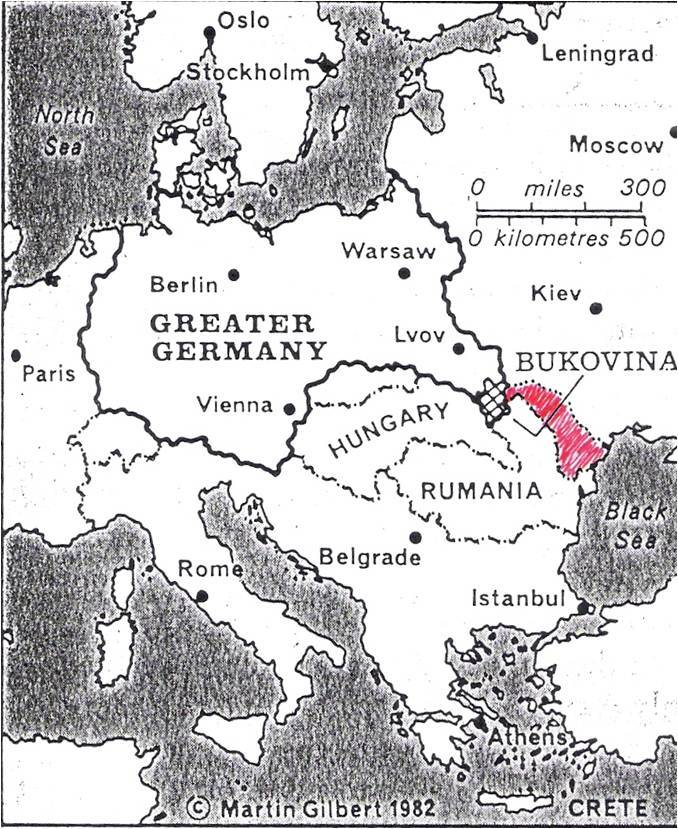
Map of Eastern Europe Showing the Location of Transnistria (red).

Born in Czernowitz in 1903, Arthur Kessler (Figure 1) only became a Romanian citizen after World War I. With a doctorate in medicine from Vienna, Kessler was recruited into the Romanian army. He was transferred to reserve duties only in 1930 and allowed to practice privately. With the Nazi invasion of Romania, his activities were progressively curtailed, and in 1942 Kessler was deported to the province of Transnistria, a narrow strip of land situated between Moldova in the west and the Ukraine in the east, between the rivers Bug and Dniester (Figure 2).^9^

### Vapniarka and Grass Pea

This region was uncultivated at the time and prone to harsh winters. The passing German forces found large quantities of grass pea horse-fodder left behind by the retreating Soviets. Possibly as a deliberate experiment^10^ they suggested feeding grass pea to the prisoners in Vapniarka. The Nazis would not have been unaware of the toxicity of grass pea (*Lathyrus sativus*), which is now known to contain a toxic alanine product known as oxalyldiaminopropionic acid (ODAP). The leadership of Vapniarka was Romanian; hence their willingness to feed their prisoners with bread baked from barley and chopped straw and grass peas boiled in salty water is noteworthy, since without doubt they also knew the grass pea was toxic—grass pea was not included in the diets of the camp personnel at all.^11^

Consumed in large quantity and over a prolonged period of time, this “nutrient” had been known to be neurotoxic since antiquity. In Europe, consuming grass pea was forbidden by George, Duke of Württemberg, in 1671, an order re-enforced by his successor, Leopold.^12^ The consumption of grass pea was also forbidden in France and Algeria in the early nineteenth century. This type of food-poisoning all but disappeared in Europe, except during the civil war in Spain and in the Balkans during WWII, but remained endemic in underdeveloped East African and Far East Asian countries. Nevertheless, historically, famine-afflicted populations relied on the grass pea when it was the only source available for nutrition.

### Detection of Grass Pea Poisoning

Kessler ended up in the Vapniarka detention center where he served as a physician to the other incarcerated prisoners. Shortly after his arrival he recorded:

… within months, hundreds of young male inmates of the camp began limping and had begun to use stick-crutches to propel themselves about, in some cases inmates had been rapidly reduced to crawling on their back sides to make their ways through the compound.^9^,^13^

Kessler collected the data of hundreds of patients who developed spastic paralysis of the lower limbs while in Vapniarka (Figure 3). He connected their condition with a never-seen and non-existent disease in Europe, not taught in medical schools and not appearing in textbooks. The symptoms involved a gradual paralysis ascending from the lower limbs to the thoracic level of the cortico-spinal tract, later on histologically proven to be a spinal cord degenerative process.^11^ This upper neuron disease, which would later be called lathyrism, appeared clinically as monoplegia, paraparesis or paraplegia, and incontinence; he meticulously recorded his findings on any available piece of paper.^15^ Kessler eventually determined that the toxic dosage of grass pea was 300 mg/day over a 3-month period (Figure 4). Kessler wrote: “We are eating poison and we will die of it.”^15^

**Figure 3:**
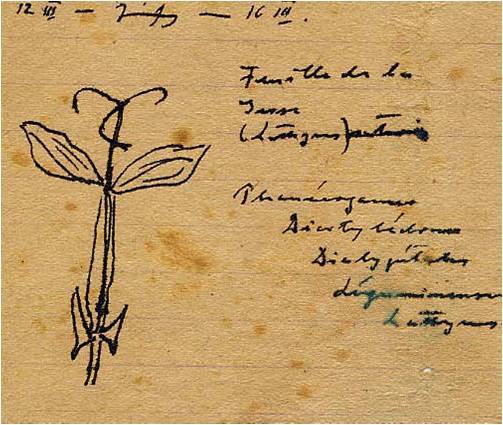
Dr Kessler’s Drawing of a Paretic Limb, Vapniarka, 1942. Source: D. Kessler,^14^ with permission.

**Figure 3:**
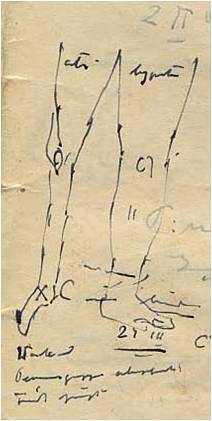
Dr Kessler’s Drawing of the Lathyrus Plant, Vapniarka, 1942. Source: D. Kessler,^14^ with permission.

Armed with the documented poisoning, the inmates organized a hunger strike.^16^ When the group confronted one of the brutal commandants of the camp, Kessler wrote that he responded, “what makes you think that we are interested in keeping you alive?”^16^

Kessler courageously persisted in confronting successive visiting authorities, some interested Romanian physicians, the Governor of the province, and a Ukrainian neurologist familiar with the disease. As a result, the toxic feeding program was replaced with a meagre food supply (two slices of bread a day, dried “fruit,” and artificially sweetened tea).^17^,^18^

### Additional Medical Challenges in Vapniarka

Despite the success in ending this toxic diet, there was no lack of patients. Starvation and the freezing Ukrainian winter led to aggravated gangrene, necessitating amputations and prostheses. Kessler also had to treat patients severely debilitated by tuberculosis, the typhus exanthematicus that flourished in the summer, despair-induced depression, and those who suffered the irreversible effects of the grass pea food-poisoning. A crutch support was improvised by the inventive physician, bandages for skin ulcerations and gangrene (angio-lathyrism) were manufactured, and various nappy supports were supplied to incontinent patients. Vascular occlusion occurred in adults, and the softened bone deformity (osteo-lathyrism) appeared in developing skeletons.^11^,^13^,^15^,^17^–^20^

### Post-Vapniarka

The camp was eventually dismantled, and Kessler was transferred to Olgopol, another camp in the province with even worse conditions. The hunger was more severe, the tuberculosis was more advanced, and typhus was more prevalent. When the war turned against the Nazis, the camp was closed, and the surviving prisoners were returned to Romania proper. There were hundreds of camps in that region, yet today almost nothing remains to testify of their existence, and residents of the region deny that Vapniarka ever existed.^17^

Kessler was fortunate to be amongst the surviving half of the 800,000 Jews who lived in pre-war Romania. His documents were hidden and published after liberation.

After the war, Kessler immigrated to Israel and was employed by one of the health maintenance organizations there, practicing allergology. Kessler published numerous articles in local and international medical journals, focusing on lathyrism and pediatric allergology.^9^ His allergy-related research spanned a number of topics, including bee stings, allergies in infants, sensitivity to medications, bronchial asthma, and allergies to house dust, to mites, and to birds. He warned of the risk of aerosols and dealt with the influence of Israel’s climate on asthma patients, including publications on asthma mortality. Many of his publications remain relevant and continue to be cited today.

### Kessler’s Legacy

Kessler’s research was foundational to the discovery of and subsequent research on the residual neural, vascular, and osteopathic symptoms and findings of lathyrism. Lathyrism would continue to be extensively investigated in Ichilov Hospital, Tel Aviv, particularly in the 200 survivors of Vapniarka.^13^,^15^,^19^,^20^

Dr Arthur Kessler died in Israel in 2000. However, his research and toxicology tables remain in use to this day. In one renowned case, knowledge of his research led to determining the “mysterious” cause of death of a college student in Alaska.^21^ Kessler would have been pleased to know that today a new detoxified grass pea that is alanine toxin-free and ODAP-free is being cultivated for use in famine-hit areas.^22^

## BRONISLAWA FEJGIN

Bronislawa Fejgin was a physician who was murdered during the Shoah. Despite her cut-short career, the legacy of her research into bacteriology and serology is of ongoing relevance.^23^

**Figure 5:**
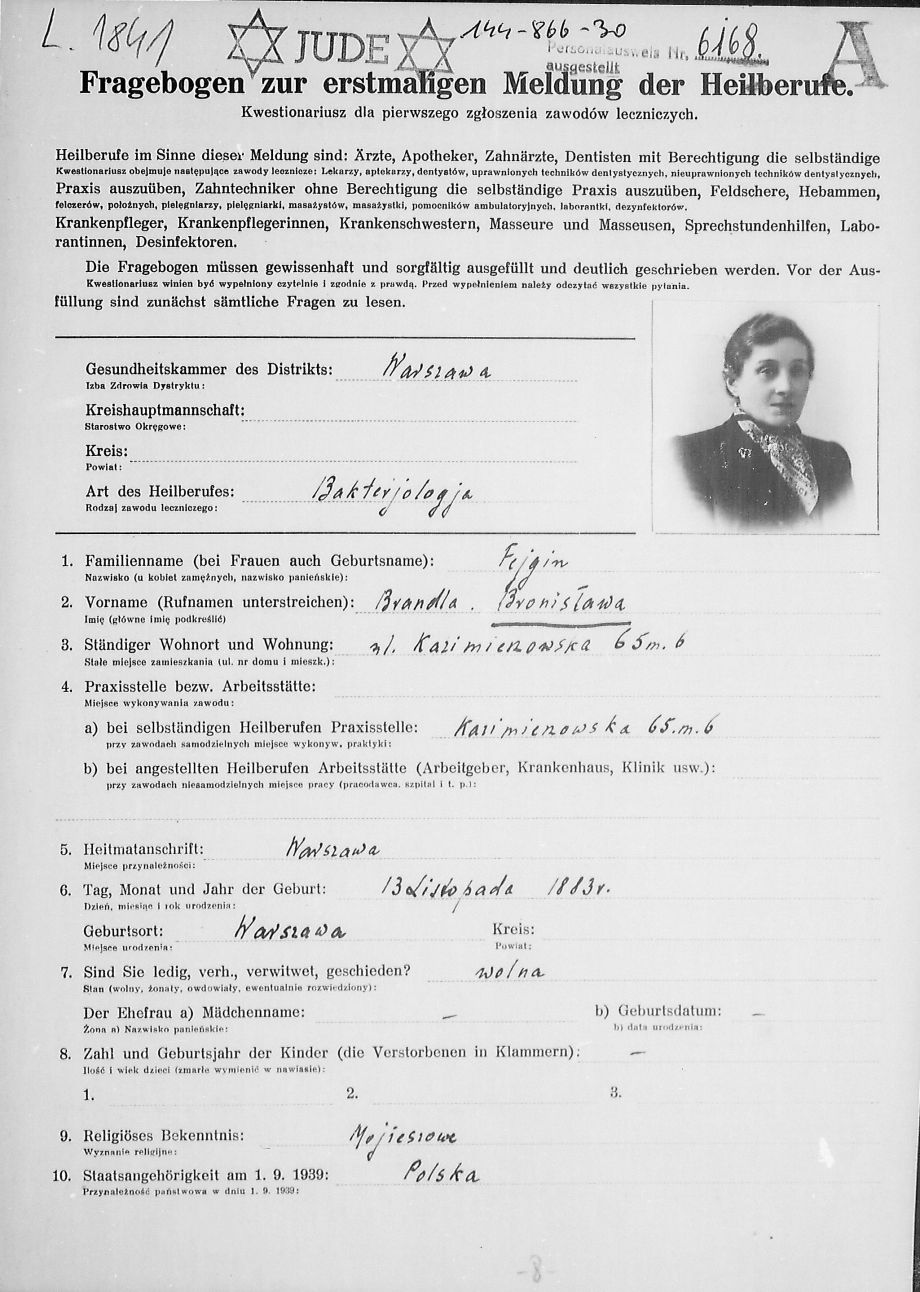
Dr Bronislawa Fejgin’s Warsaw Ghetto Registration Document. Source: Main Physicians Library, Warsaw, Poland (public domain).

Her brief biography would tell the following short story: Fejgin was born in Warsaw in November 1883, a date that appears on her diploma issued by the Sorbonne Medical School in Paris in 1914, as well as her admission form to the Warsaw Ghetto on October 9, 1940 (Figure 5). There is almost no information about her family or her life prior to university.

After graduation, Fejgin returned to Warsaw where she rapidly advanced and eventually headed the State Institute of Hygiene. Apart from local journals, her discoveries were published in French and English and even some German literature. In 1940, after being incarcerated in the Warsaw Ghetto, she became manager of the Bacteriological Institute there.

Fejgin also taught bacteriology in the clandestine Medical School that the Czyste Jewish Hospital staff conducted in the ghetto under the disguise of a technical school for the prevention of infectious diseases. The school was never discovered and lasted some 10 months before mass deportation to Belzec.^1^ This exceptional and ingenious effort by the Jewish authorities in the ghetto involved organizing nighttime lectures for some 450 students deported to Warsaw from all over Poland. Preclinical lectures were offered, covering topics such as anatomy, histology, physiology, bacteriology, and clinical care.^8^

The lectures were taught under lantern or candle light, microscopes were smuggled in by sympathetic Polish (former) colleagues, and exams were recorded and final marks assessed. The hope of surviving and continuing medical practice in the Jewish community was the driving force of the teachers. The results of the exams were buried in metal containers and exhumed after the war. The surviving 50 students had their examinations recognized, and they were able to complete their medical studies.^8^

### Scientific Contributions

Fejgin’s scientific contributions impacted three different lines of bacteriology and serology, which at times overlapped. They are briefly reviewed below by topic rather than in chronological order.

#### General bacteriology

Fejgin’s doctoral thesis in Paris was on bacteriology and vaccinations for the inflamed uterus. She also studied diphtheria and isolated a batch of filterable anti-diphtheria fluid (lytic form) that could be produced in guinea-pigs or rabbits.^23^ This was the early form of what would later appear as the protective bacteriophage of Twort–D’Herelle.^24^,^25^

One of her important epidemiological discoveries related to the transmission of scarlet fever. Fejgin discovered that if fingers that had been infected with streptococcus by saliva were used to turn the pages of books the bacteria remained active on the pages for 4–6 weeks, rendering the pages infectious to the next reader.^26^

#### Typhus exanthematicus (spotted fever)

The second line of interest is revealed through Fejgin’s numerous published results diagnosing typhus exanthematicus (spotted fever). She was the discoverer of cross-agglutination between Proteus HX 19 bacillus and the typhus “virus,” later identified as Rickettsia prowazeki. By reproducing a filterable form of HX 19 and injecting guinea-pigs intraperitoneally, a milder form of typhus fever was obtained, known in 1922–1925 as Nicolle disease.^27^,^28^

Hers was a basic study for future preparation of vaccines. Equally important was a diagnostic test that she developed: Fejgin found that the urine of typhus patients agglutinated a proteus antigen, a test useful for identifying typhus when no serum was available.^28^ Fejgin stated that in 1908 she established the presence of agglutinants to intestinal bacilli in the serum of typhus fever patients. This essential test was later utilized for serological diagnosis of typhus exanthematicus by Rudolf Weigl in Poland in the 1920s and 1930s and by Ludwik Fleck in the Lwów Ghetto.^6^

#### Phage phenomenon

The third line of interest in Fejgin’s research related to the recently introduced phage phenomenon, described by Twort in 1915 and by D’Herelle in 1917. Their findings were actually based on Fejgin’s, namely that the phenomenon of bacterial autolysis resulted from an intracellular lytic agent, a “virus,” which was produced by the bacteria itself and would later become known as a bacteriophage.^23^ Her studies also complemented those of Twort and D’Herelle via her proposed preparation of the lytic agent for Proteus HX 19 and diphtheria.^23^–^27^

Fejgin expanded the bacteriophage studies on shigella, diphtheria, typhoid, and proteus, all with cultures, as well as agglutination and antibacterial lytic agent production that led to a new species of bacteria with diminished virulence. In 1922 she concluded that the lytic agent was produced by live bacteria and is lytic only to live bacteria.^28^ This proved to be an essential and basic concept for phage therapy.

There has been a prolonged controversy in the literature regarding the true discoverer of the bacteriophage. Based on the sequence of Fejgin’s publications, she made a very early and essential contribution.^29^–^31^ By the end of the 1930s and the early 1940s bacteriophage research had markedly decreased, if not entirely been abandoned, partly because of the emergence of antibiotics, but perhaps as well due to Fejgin’s untimely demise in the Warsaw Ghetto in January of 1943.^1^,^5^

### Fejgin’s Legacy

Today, bacteriophage therapy is emerging as an essential in the fight against antibiotic-resistant bacteria. Despite dozens of publications, of which only a few are cited herein, her name is not to be found in a MEDLINE search, a fact that should be rectified. Clearly Fejgin was an anomaly in the male-dominated world of medical research; this too may be a factor in her contributions, for the most part, going unrecognized.^32^,^33^

## CONCLUSION

In conclusion, it seems appropriate to recall the statement of Dr Milejkowski:

You too [Drs Kessler and Fejgin], were part of the whole, You too were menaced by forced labour, starvation, deportation, by all the forms of death that stalked our Ghetto. And you gave the murderers a bold answer with your work: I shall not die in vain.^8^

## Abbreviations

ODAP: oxalyldiaminopropionic acid
